# Recent advances in understanding inherited disorders of keratinization

**DOI:** 10.12688/f1000research.14514.1

**Published:** 2018-06-27

**Authors:** Theodore Zaki, Keith Choate

**Affiliations:** 1 Department of Dermatology, Yale University School of Medicine, New Haven, Connecticut, USA; 2 Department of Genetics, Yale University School of Medicine, New Haven, Connecticut, USA; 3 Department of Pathology, Yale University School of Medicine, New Haven, Connecticut, USA

**Keywords:** Ichthyosis, Corneocyte lipid envelope, keratinization disorders

## Abstract

The ichthyoses are a heterogeneous group of skin diseases characterized by localized or generalized scaling or both. Other common manifestations include palmoplantar keratoderma, erythroderma, recurrent infections, and hypohidrosis. Abnormal barrier function is a cardinal feature of the ichthyoses, which results in compensatory hyperproliferation and transepidermal water loss. Barrier function is maintained primarily by the stratum corneum, which is composed of cornified cells surrounded by a corneocyte lipid envelope and intercellular lipid layers. The lipid components are composed primarily of ceramides. Human genetics has advanced our understanding of the role of the epidermal lipid barrier, and a series of discoveries in animals and humans revealed mutations in novel genes causing disorders of keratinization. Recently, next-generation sequencing has further expanded our knowledge, identifying novel mutations that disrupt the ceramide pathway and result in disorders of keratinization. This review focuses on new findings in ichthyoses caused by mutations involving lipid synthesis or function or both.

## Introduction

The ichthyoses are a heterogeneous group of skin diseases characterized by localized or generalized scaling or both. Other common manifestations include palmoplantar keratoderma (thickening of palms and soles), erythroderma (reddening of the skin), recurrent infections, and hypohidrosis (diminished sweating). Abnormal barrier function is a cardinal feature of the ichthyoses, which results in compensatory hyperproliferation and transepidermal water loss.

**Figure 1.**
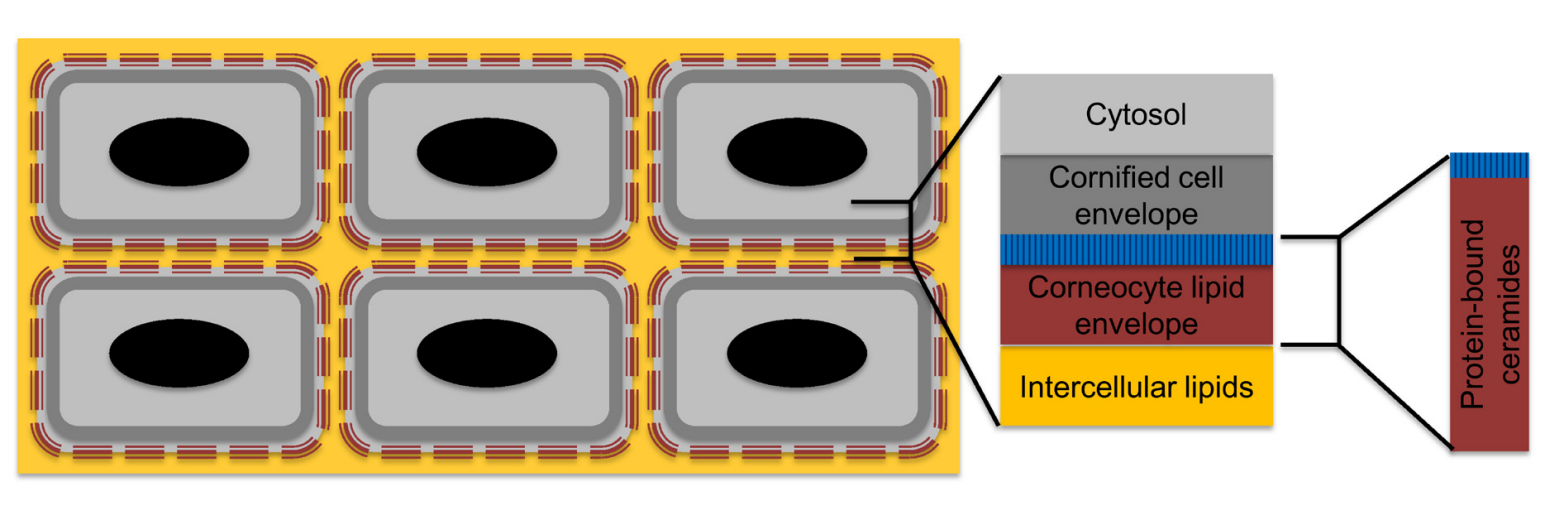
Components of the stratum corneum. The stratum corneum is composed of the corneocytes surrounded by the cornified cell envelope, the corneocyte lipid envelope spanned by protein-bound ceramides, and the intercellular lipid layer. Acylceramides are produced primarily in cells of the stratum granulosum and the stratum spinosum and are stored in lamellar bodies as glucosylated forms. These lamellar bodies fuse with the plasma membrane at the interface of the stratum granulosum and stratum corneum, releasing the glycosylated acylceramides into the extracellular space, where they are converted to acylceramides. The released acylceramides combine with cholesterol and fatty acids to form the lipid lamellae in the stratum corneum. Some acylceramide is hydrolyzed to ω-hydroxyceramide and covalently binds to the cornified cell envelope to create corneocyte lipid envelopes.

Mutations in over 50 genes have been reported to cause syndromic and non-syndromic ichthyoses, affecting keratinocyte proteins (“bricks”); lipid metabolism, assembly, and/or transport (“mortar”); cell–cell junctions; and DNA transcription and repair ^1^. Each of these mutations results in a disruption of barrier function. The barrier function of the epidermis is maintained by site-specific expression of proteins that results in a regulated differentiation pattern as cells travel from the innermost stratum basale to the outermost stratum corneum. The robust stratum corneum is composed of cornified cells (corneocytes) that serve as building blocks, the cornified cell envelope, the corneocyte lipid envelope (CLE) that surrounds the corneocytes, and the intercellular lipid layers that serve as a mortar linking the corneocytes ( Figure 1). The corneocytes are composed of keratin, filaggrin, and their degradation products; the CLE and the intercellular lipid layers are composed primarily of ceramides (but also other lipids such as cholesterol and triglycerides) secreted by keratinocytes ^2^. Ceramides have long been known to play a role in keratinization; of the major ceramides identified to date, most have been found in the stratum corneum ^3^. Ceramides have also recently been shown to play a role in the proliferation and differentiation of epidermal keratinocytes ^4^.

**Figure 2.**
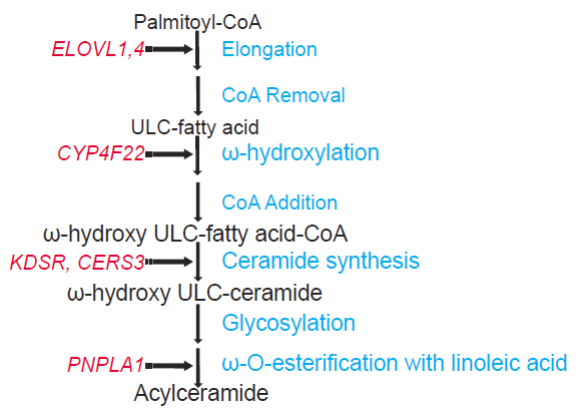
The pathway of acylceramide synthesis in keratinocytes. Key enzymes whose deficiencies are known to cause disorders of keratinization are in red and are designated by dotted arrows. CERS3, ceramide synthase 3; CYP4F22, cytochrome P450 family 4 subfamily F member 22; ELOVL, elongation of very long chain fatty acids-like; KDSR, 3-ketodihydrosphingosine reductase; PNPLA1, patatin-like phospholipase domain-containing protein 1; ULC, ultra-long-chain.

Genetic investigation has informed our understanding of the role of epidermal ceramides in lipid function and ichthyosis pathogenesis. Linkage analysis permitted positional cloning of a series of genes relevant to epidermal barrier function. Mutations in *CYP4F22* were identified as causative for autosomal recessive congenital ichthyosis (ARCI) in 2006 ^5^ and have recently been shown to disrupt ω-hydroxylation of ultra-long-chain (ULC) fatty acid for ceramide production ^6^. Mutations in *CERS3* have been shown to disrupt ceramide synthesis, resulting in ARCI ^7, 8^. More recently, next-generation sequencing has been used to identify mutations in *ELOVL4* as causative for a syndrome of ichthyosis, intellectual disability, and spastic quadriplegia by disrupting fatty acid elongation ^9^. Next-generation sequencing has been employed in disorders with small kindreds or impaired reproductive fitness to identify additional genetic causes of these disorders, finding novel mutations that disrupt the ceramide pathway ( Figure 2). This review highlights these recent findings.

## Recent advances in ichthyosis

### Mutations in *KDSR* cause recessive progressive symmetric erythrokeratoderma and thrombocytopenia

In 2017, Boyden *et al*. reported that mutations in *KDSR* (3-ketodihydrosphingosine reductase) led to a previously undescribed recessive Mendelian disorder in the progressive symmetric erythrokeratoderma spectrum—also known as periorificial and ptychotropic erythrokeratoderma (PERIOPTER) syndrome ^10^—characterized by severe lesions of thick scaly skin on the face and genitals and thickened, red, scaly skin on the hands and feet ^11^. Immunohistochemistry and yeast complementation studies have demonstrated that these mutations cause defects in KDSR function. Systemic isotretinoin therapy achieved nearly complete resolution in the two probands in whom it had been applied, consistent with the effects of retinoic acid on alternative pathways for ceramide generation.

*KDSR* mutations have been implicated in the pathobiology of hereditary palmoplantar keratodermas and ichthyosis ^11^; another recent study has demonstrated the important role that *KDSR* plays in platelet biology ^12^. *KDSR* encodes KDSR, which catalyzes the reduction of 3-ketodihydrosphingosine (KDS) to dihydrosphingosine (DHS), a key step in the ceramide synthesis pathway. The role of ceramides in platelet function is less understood, but the most likely pathomechanism for the thrombocytopenia is diminished sphingosine-1-phosphate (S1P) synthesis. This signaling lipid has been shown to promote platelet shedding from megakaryocytes ^13^, and other studies have demonstrated that exogenous S1P and ceramides can restore platelet secretion and aggregation in knockout mice deficient in S1P and ceramides ^14, 15^. While *KDSR* mutations block *de novo* ceramide biosynthesis, retinoids induce the salvage pathway for ceramide synthesis, providing pathogenesis-directed therapy of skin disease in some subjects.

### Mutations in *PNPLA1* cause autosomal recessive congenital ichthyosis by disrupting acylceramide biosynthesis

In 2012, Grall *et al*. found that mutations in the patatin-like phospholipase domain-containing protein 1 ( *PNPLA1*) gene cause ARCI in dogs and humans via a positional cloning approach ^16^. The phenotypic spectrum of *PNPLA1* mutations is broad and can include a collodion membrane at birth; mature phenotypes can include fine or plate-like scale and erythema that can range from minimal to severe ^17^.

Recent studies in cell-based and *in vitro* assays have shown that PNPLA1 is directly involved in acylceramide synthesis as a transacylase, catalyzing ω-O-esterification with linoleic acid to produce acylceramide ^18^. In PNPLA1 knockout mice, loss of ω-O-acylceramides in the stratum corneum results in a defective CLE and a disorganized extracellular lipid matrix ^19– 21^. The administration of topical acylceramide on the skin of PNPLA1-deficient mice was shown to rebuild the CLE, partially rescuing the ichthyosis phenotype ^19, 21^.

### Mutations in *SDR9C7* cause autosomal recessive congenital ichthyosis

In 2016, Shigehara *et al*. described a homozygous missense mutation in short-chain dehydrogenase/reductase family 9C member 7 (SDR9C7) underlying ARCI in three consanguineous Lebanese families and showed that SDR9C7 is expressed in the granular and cornified layers of the epidermis ^22^. The pathomechanism of ichthyosis caused by SDR9C7 deficiency has been debated. Shigehara *et al*. cited prior evidence of SDR9C7 converting retinal into retinol ^23^, suggesting that the ichthyosis phenotype results from a vitamin A deficiency impairing epidermal differentiation ^22^. Takeichi *et al*. noted reduced lipid contents and defective intercellular lipid layers in the stratum corneum on electron microscopy and postulated that the pathomechanism of the ichthyosis phenotype in SDR9C7 deficiency involves defective synthesis and metabolism of keratinocyte lipid contents ^24^.

### Mutations in *ELOVL1* cause neurological disorder with ichthyotic keratoderma, spasticity, hypomyelination, and dysmorphic features

In 2018, Kutkowska-Kaźmierczak *et al*. described a dominant missense mutation in elongation of very long chain fatty acids (VLCFAs)-like 1 ( *ELOVL1*) in two kindreds that resulted in a syndrome of ichthyotic keratoderma, spasticity, mild hypomyelination, and dysmorphic features ^25^. Like ELOVL4, ELOVL1 is involved in fatty acid elongation, catalyzing the synthesis of saturated and mono-unsaturated VLCFAs ^26^. ELOVL1 activity has also been shown to be regulated with the ceramide synthase CERS2, which is essential for C24 sphingolipid synthesis ^27^. A prior murine model deficient in *Elovl1* demonstrated wrinkled, shiny, red skin, and electron microscopy showed diminished lipid lamellae in the stratum corneum. Thin-layer chromatography revealed decreased levels of ceramides with ≥C _26_ fatty acids ^28^. Kutkowska-Kaźmierczak *et al*. suggest that the disease may result from the shortage of VLCFAs due to the lack of activity of mutated enzymes and speculate that the mutation may have a greater impact on VLCFA levels in the brain and skin than in fibroblasts or plasma ^25^.

## Abbreviations

ARCI, autosomal recessive congenital ichthyosis; CLE, corneocyte lipid envelope; ELOVL, elongation of very long chain fatty acids-like; KDSR, 3-ketodihydrosphingosine reductase; PNPLA1, patatin-like phospholipase domain-containing protein 1; S1P, sphingosine-1-phosphate; SDR9C7, short-chain dehydrogenase/reductase family 9C member 7; VLCFA, very long chain fatty acid.
